# A drastic post operative course after the resection of primary pulmonary choriocarcinoma in a male

**DOI:** 10.1002/rcr2.1400

**Published:** 2024-07-01

**Authors:** Makoto Takahama

**Affiliations:** ^1^ Department of General Thoracic Surgery Osaka City General Hospital Osaka Japan

**Keywords:** male, primary pulmonary choriocarcinoma, tumour emboli

## Abstract

Primary pulmonary choriocarcinoma is a highly aggressive germ cell neoplasm and an extremely rare, especially in males. It is characterized by a poor response to therapy and shortened survival times. We present the case of primary pulmonary choriocarcinoma in a 46‐year‐old male. The patient was referred to our institute with cough, worsening dyspnea and hemoptysis. The contrast‐enhanced chest computed tomography revealed an avid enhanced 15 × 14 cm sized nodular lesion, in the left lower lung, which invaded into the diaphragm. After the embolization of the intercostal arteries, the tumour was resected successfully. However, the patient had died suddenly on the 28th day after the surgery. Autopsy was conducted and revealed that his cause of the death was the tumour emboli in the right coronary artery.

## Graphical abstract

A notable aspect of this case is that the primary pulmonary choriocarcinoma initially mimicked chronic expanding hematoma radiographically. The discussion of this case, the treatment course, and outcome highlight the potential challenge in correctly diagnosing primary pulmonary choriocarcinoma as well as its extremely poor prognosis in the absence of an effective, standardized treatments.

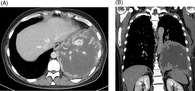

## INTRODUCTION

Primary pulmonary choriocarcinoma (PPC) in a male is an extremely rare and highly malignant germ cell tumour that secretes human chorionic gonadotropin (hCG) β‐subunit and arises spontaneously in the lung the clinical entity can be easily misdiagnosed or become a delayed diagnosis due to its unspecific clinical characteristics.^1^ Due to its rarity, few data are available on PPC, and fewer than 70 cases have been reported.^2^ We here present a case of PPC in 46‐year‐old male initially mimicking chronic expanding hematoma radiographically.

## CASE REPORT

**FIGURE 1:**
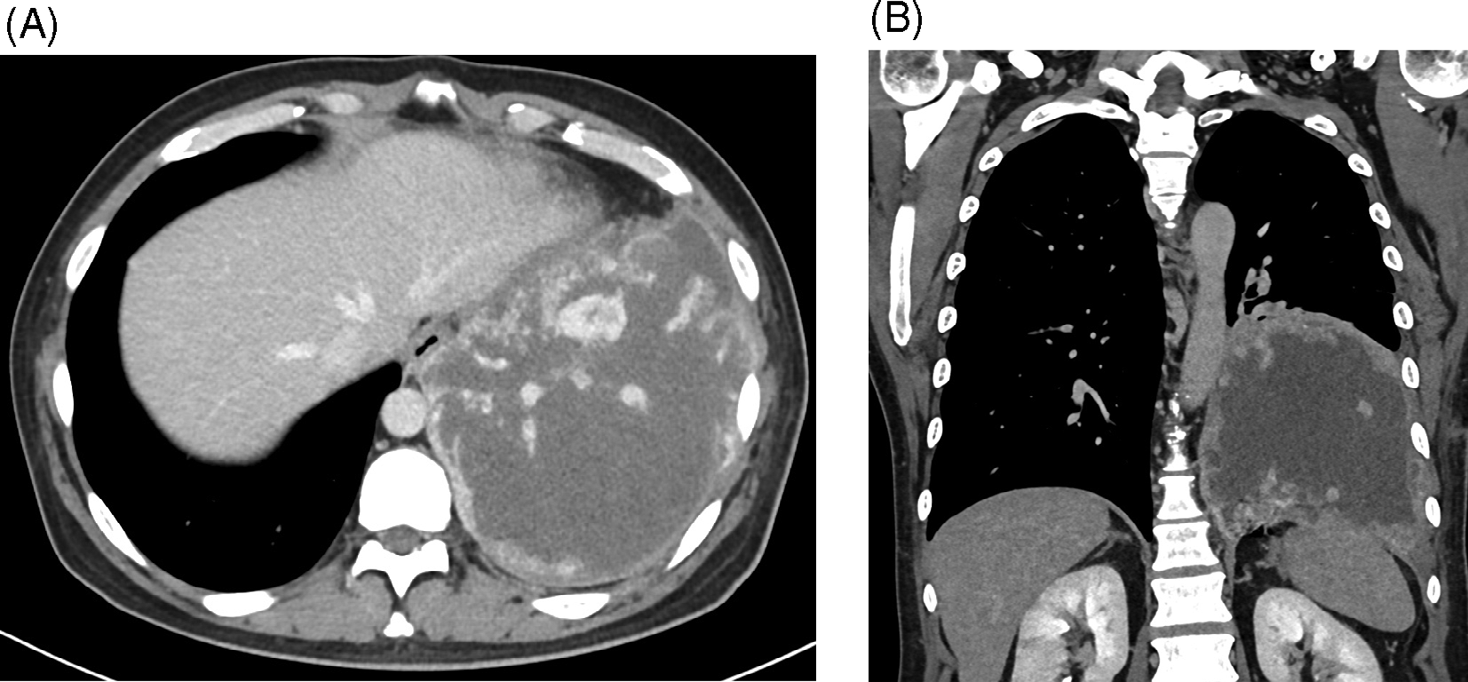
(A) Chest axial image computed tomography (CT) demonstrated 15 × 14 cm sized mass in the left lower lobe in the lung. (B) Coronal CT images. The circumference of the mass showed a high contrast enhancement similar to that of blood vessel.

**FIGURE 2:**
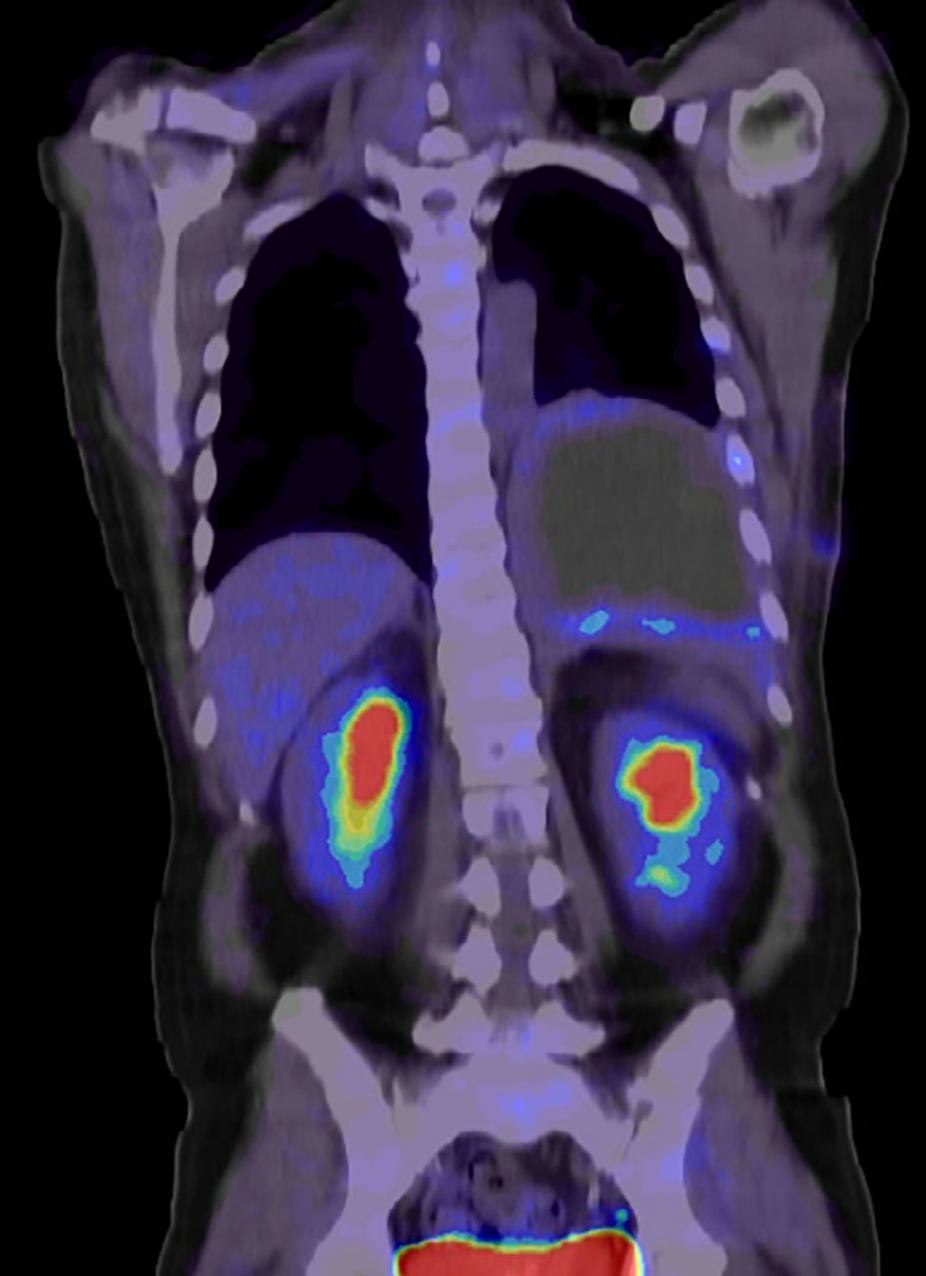
^18^F‐Fluorodeoxyglucose (FDG)‐positron emission tomography scan. FDG uptake was detected heterogeneously in the circumference of the mass. Standardized uptake value max was 5.5.

A 46‐year‐old male with no history of cigarette smoking was referred to our institute with cough, worsening dyspnea and hemoptysis. He received a medication for the treatment of diabetes mellitus. Chest contrast‐enhanced computed tomography (CT) revealed highly enhancing mass lesion measuring approximately 15 × 14 cm in the left lower lobe of the lung, which invaded to the diaphragm (Figure 1A). The mass was enhanced heterogeneously with necrosis in the left lower lobe of the lung and the circumference of the mass showed contrast enhancement similar to that of blood vessels (Figure 1B). F‐18 fluorodeoxyglucose (FDG)‐positron emission tomography scan revealed that FDG uptake was detected in the circumference of the mass only (Figure 2). The initial differential diagnosis of this mass was chronic expanding hematoma in the pleura radiographically. The results of the routine laboratory examinations were all normal. Because we could not deny completely that this mass was not a malignant lung tumour, we examined serum Cyfra level. The serum Cyfra level elevated to 15.0 ng/mL (normal range, <3.5 ng/mL). The preoperative β‐hCG levels in the serum and urine were not examined. A histological examination of the mass was avoided due to the risk of haemorrhage. In the absence of distant metastasis and regional metastases, the patient underwent left lower lobectomy and the resection and reconstruction of the diaphragm after the embolization of the intercostal arteries to reduce the intraoperative bleeding.

The macroscopic finding consisted of a large haemorrhage mass measuring 16 × 13.5 cm occupying more than half of the resected lung with necrosis and a haemorrhage adjacent to the lung parenchyma.

**FIGURE 3:**
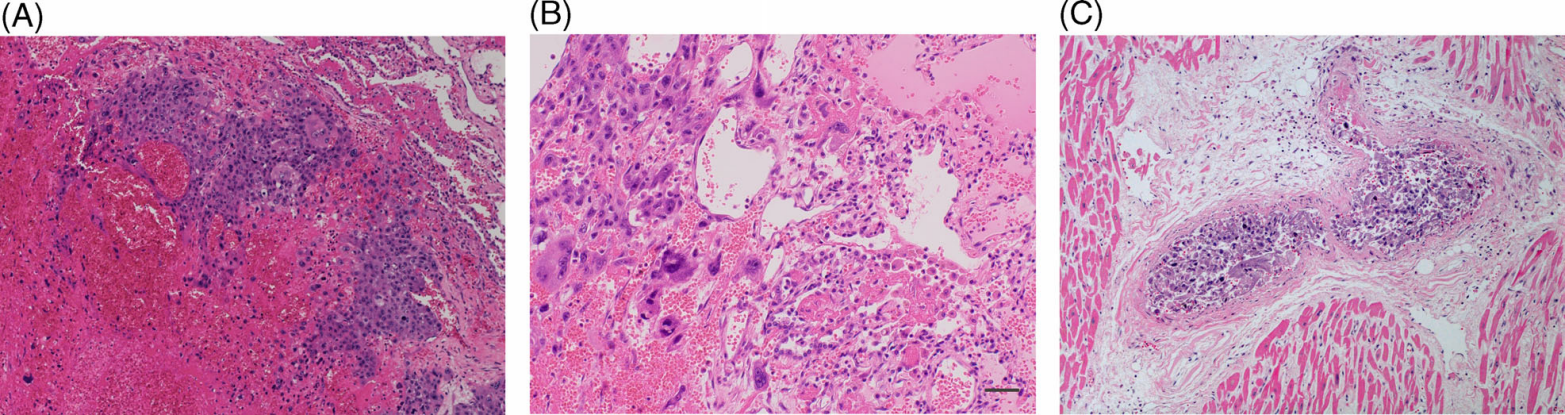
(A) Haematoxylin and eosin (H&E) stain of the left lung tumour showed sheets of highly pleomorphic tumour cell with haemorrhage and necrosis (×100). (B) Higher magnification of tumour revealed a large hyperchromatic multinucleated syncytiotrophoblasts and surrounding mononuclear cytotrophoblasts and intermediate trophoblasts (H&E, ×200). (C) The lumens of the vessel were completely occupied by the tumour cells (H&E, ×100).

Pathologic findings of the resected mass revelled that the tumour cell were poorly differentiated and had a characteristic biphasic pattern with polygonal cytotrophoblastic cells growing in a nest‐like fashion that were separated or capped by multinucleated giant syncytiotrophoblastic cells (Figure 3A,B). The tumour had invaded into the diaphragm, but did not exhibit lymph node involvement; the surgical resection margins were clear. Immunohistochemical examination revealed staining for β‐hCG was strongly positive and for CKAE1/3 was positive in the tumour cells. Staining for Glypican3, SALL4, p40 and CD31 was weakly focally positive in the tumour cells. Staining for CD30, TTF‐1, D2‐40 and ERG was negative. Based on these findings, the diagnosis of choriocarcinoma was confirmed. After the operation, the serum β‐hCG level was measured for the first time. The serumβ‐hCG was 5906.0 mIU/mL (normal, 0–2 mIU/mL).

Unfortunately, the patient had died suddenly on the 28th day after the surgery during sleeping. Autopsy was conducted and revealed that the tumour cells were detected in the right coronary artery (Figure 3C). Therefore, his cause of the death was diagnosed the acute myocardial infarction due to the tumour emboli in the right coronary artery.

## DISCUSSION

Primary extragenital choriocarcinoma is rare, usually presenting as a midline lesion in the retroperitoneum, mediastinum, or intracranium. In men, choriocarinoma occurs most often in the testis. Less frequently, these tumours have also been reported in various organs, such as the urinary bladder, liver, stomach and colon.^3^ It has been hypothesized that the retained primordial germ cells may migrate abnormally during embryonic development into the above‐mentioned locations and development into choriocarcinoma.^2^ Among such sites, PPC is extremely rare. Because the lung is a frequent site of metastasis for choriocarcinoma, a careful examination for an occult primary tumour is required through preoperative diagnosis of PPC is difficult.

In the present case, because the preoperative histological examination and β‐hCG level was absent, the diagnosis of PPC was based on the histological features combined with immunohistochemical detection of the resected tumour.

The clinical characteristics of PPC vary and usually present symptoms such as dyspnea, cough, chest pain or hemoptysis. In men, signs of feminization, such as gynecomastia, loss of libido, and testicular atrophy, may be seen, which are associated with elevation of β‐hCG levels. In the present case, preoperative CT scan revealed that the patient had gynecomastia (data not shown), however we did not recognize this phenomenon during a preoperative period.

Extragonadal primary choriocarcinoma in males is aggressive with a much poorer prognosis than other histological subtypes most likely related to the hematogenous dissemination.^4^ The treatment strategies performed for the patients of PPC included complete resection, chemotherapy, radiotherapy, or a combination of the above treatment.^1^, ^4^ At present, there is no established standard therapy for PPC, which is mainly administrated according to the treatment guidelines for choriocarcinoma and the clinicians' experience.^2^ Nevertheless, the patients of PPC had a higher metastatic rate and poorer survival rate. To explore the optimal treatment for PPC, a univariate and multifactorial analysis of the data collected was carried out, showing that chemotherapy alone seldom improved the prognosis of patients, but surgery followed by adjuvant chemotherapy had a remarkable effect on the prognosis and was independently prognostically significant.^2^ Therefore, adjuvant chemotherapy after surgery seems to be the best treatment for PPC.

In conclusion, PPC in a male is extremely rare. The prognosis of PPC is extremely poor, despite surgical and chemotherapeutic treatment. Currently, there is no standard treatment for PPC. Signs of feminization would be helpful to diagnose as PPC.

## AUTHOR CONTRIBUTIONS

MT drafted manuscript and have read and approved the final version of the manuscript.

## CONFLICT OF INTEREST STATEMENT

None declared.

## ETHICS STATEMENT

This case report was approved by the Osaka City General Hospital Institutional Review Board and the patient provided written informed consent for the use of these data.

## INFORMED CONSENT STATEMENT

Written informed consent obtained from the patient for the publication of this manuscript and associated images.
